# Cost-effectiveness analysis of different systolic blood pressure targets for people with a history of stroke or transient ischaemic attack: Economic analysis of the PAST-BP study

**DOI:** 10.1177/2047487316651982

**Published:** 2016-05-25

**Authors:** Maria Cristina Penaloza-Ramos, Sue Jowett, Pelham Barton, Andrea Roalfe, Kate Fletcher, Clare J Taylor, FD Richard Hobbs, Richard J McManus, Jonathan Mant

**Affiliations:** 1 Health Economics Unit, University of Birmingham, UK; 2 Primary Care Clinical Sciences, University of Birmingham, UK; 3 Nuffield Department of Primary Health Care Sciences, University of Oxford, UK; 4 Primary Care Unit, University of Cambridge, UK

**Keywords:** Hypertension, stroke, transient ischaemic attack, blood pressure target, cost effectiveness, decision model, decision analysis

## Abstract

**Background:**

The PAST-BP trial found that using a lower systolic blood pressure target (<130 mmHg or lower versus <140 mmHg) in a primary care population with prevalent cerebrovascular disease was associated with a small additional reduction in blood pressure (2.9 mmHg).

**Objectives:**

To determine the cost effectiveness of an intensive systolic blood pressure target (<130 mmHg or lower) compared with a standard target (<140 mmHg) in people with a history of stroke or transient ischaemic attack on general practice stroke/transient ischaemic attack registers in England.

**Methods:**

A Markov model with a one-year time cycle and a 30-year time horizon was used to estimate the cost per quality-adjusted life year of an intensive target versus a standard target. Individual patient level data were used from the PAST-BP trial with regard to change in blood pressure and numbers of primary care consultations over a 12-month period. Published sources were used to estimate life expectancy and risks of cardiovascular events and their associated costs and utilities.

**Results:**

In the base-case results, aiming for an intensive blood pressure target was dominant, with the incremental lifetime costs being £169 lower per patient than for the standard blood pressure target with a 0.08 quality-adjusted life year gain. This was robust to sensitivity analyses, unless intensive blood pressure lowering reduced quality of life by 2% or more.

**Conclusion:**

Aiming for a systolic blood pressure target of <130 mmHg or lower is cost effective in people who have had a stroke/transient ischaemic attack in the community, but it is difficult to separate out the impact of the lower target from the impact of more active management of blood pressure.

## Background

Stroke is a major cause of morbidity and mortality in the UK. There are approximately 110,000 strokes per year in England and around 300,000 people living with moderate to severe disabilities as a result of stroke.^1^ After a first stroke, patients are at high risk of a recurrent event: for every 1000 first strokes, 240 will have a recurrent cardiovascular disease event within five years of the first episode, of which 180 would be a stroke and 29 of these would be fatal.^2^ In 2008–2009, the direct care cost of stroke was £3 billion annually, within a wider economic cost of about £8 billion. Without preventive action, there is likely to be an increase in strokes as the population ages.^1^ Therefore, secondary prevention has a major potential role to play in reducing both morbidity and costs of stroke care.

There is controversy over how intensively to lower blood pressure (BP) in people who have had a stroke, with different international guidelines recommending different target BPs,^3,4^ and uncertainty over the applicability of the current evidence base for BP reduction after stroke to people with a history of transient ischaemic attack (TIA) or stroke in community populations.^5,6^ A systematic review of the effect of intensive BP lowering in populations including those with a history of stroke found that more intensive BP lowering does lead to reduced risk of major cardiovascular events,^6^ and the recent SPRINT trial, albeit in a population without a history of stroke, found that intensive BP lowering reduced major cardiovascular events and all-cause mortality.^7^ Therefore, there is renewed interest in strategies to lower BP intensively in high-risk populations, such as those with a history of stroke or TIA. The Prevention AfTer Stroke – Blood Pressure (PAST-BP) randomised controlled trial compared the impact of an intensive systolic blood pressure (SBP) target (<130 mmHg or 10 mmHg reduction from baseline if this was <140 mmHg) with a standard target (<140 mmHg) in people with a history of stroke or TIA recruited from primary care.^8^ The trial involved active management in all patients, and found that this led to important reductions in BP in both arms.^9^ The more intensive target was associated with only a small additional reduction in BP (2.9 mmHg), which raises the question as to whether such an intensive target is cost effective.

Here, we report the results of a model-based cost-utility analysis, which extrapolates the results of the PAST-BP trial^9^ to estimate the long-term cost effectiveness of intensive BP lowering targets after stroke/TIA in a primary care population, compared to a standard target.

## Methods

A Markov model was constructed to estimate the long-term cost effectiveness, in terms of the cost per quality-adjusted life year (QALY) gained, of an intensive target strategy versus a standard target strategy for BP lowering in people with a history of stroke or TIA. The model was developed using TreeAge Pro Suite 2012 software (TreeAge Software Inc., Williamstown, MA, USA). The analysis was conducted from a UK National Health Service (NHS) and personal social services perspective.^10^

**Figure 1.**
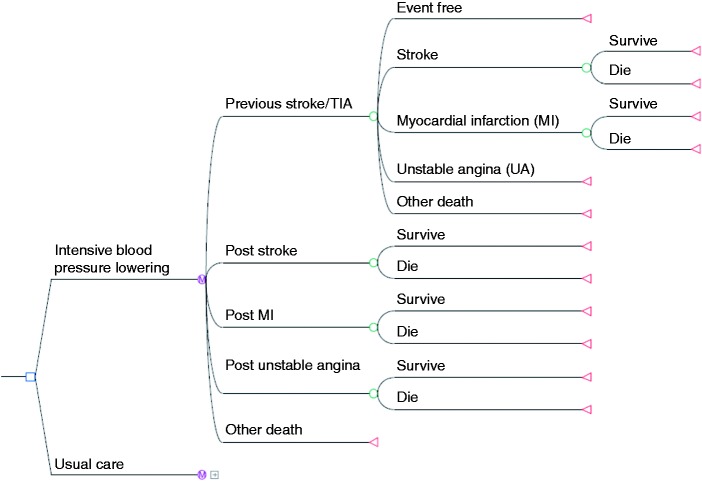
Markov model. *Note*: The Markov model in this figure is only being displayed for the ‘intensive blood pressure lowering’ strategy. The standard target strategy is identical. Similarly, the model is identical at every node ending with green circles. Final outcomes (shown as red triangles) are survival and death.

The model had a time cycle of one year with a 30-year time horizon (i.e. lifetime). The base-case analysis considered a cohort similar to that recruited to the PAST-BP trial (aged 70 years, 41% female). Baseline characteristics for important potential confounders were similar in both arms.^9^ Movements between model health states were defined by transition probabilities, which represented the risk of experiencing an event within a year time cycle. Long-term costs and health outcomes were assessed by attaching estimates of costs and utilities to the model health states. QALYs were calculated by multiplying life expectancy by the health state utility. Cost effectiveness was expressed as cost per additional QALY gained. The structure of the Markov model is shown in Figure 1.

Individual patient level data were used from the PAST-BP trial^9^ supplemented by parameter estimates from published studies (Table 1). In the PAST-BP trial^9^ participants were recruited from stroke/TIA registers in English general practices during 2009–2011 and were randomly assigned to an intensive BP target (<130 mmHg or a 10 mmHg reduction if baseline pressure was <140 mmHg) or a standard SBP target (<140 mmHg). Over one year, mean SBP dropped by 16.1 mmHg in the intensive target arm and by 12.8 mmHg in the standard arm (adjusted difference between groups 2.9 mmHg, *P* = 0.03). For extrapolation beyond one year, we assumed that this difference in BP was maintained.

**Table 1.** Model parameters.

Parameter	Value	Distribution	Source
**Reduction in systolic blood pressure at 12 months (mmHg)**			
Intensive BP lowering	16.1		PAST-BP trial^9^
Standard target	12.8		
12 Months’ difference between groups (95% CI)	–2.9 (–5.7, −0.2)		
**Annual event probabilities**			
Stroke			
60–69 years old	0.0348		PROGRESS & NICE, Lipid Modification Guidelines^12,18^
70–79 years old	0.0589		
80–89 years old	0.0713		
MI and UA			
60–69 years old	0.0139		PROGRESS & NICE, Lipid Modification Guidelines^12,18^
70–79 years old	0.0232		
80–89 years old	0.0232		
**Age-related relative risks at 12 months for intensive and standard BP lowering** ^a^			
MI and UA – intensive BP lowering			
60–69 years old	0.62 [0.59, 0.65]		PAST-BP trial & Law et al.^9,13^
70–79 years old	0.68 [0.63, 0.70]		
80–89 years old	0.74 [0.69, 0.77]		
Stroke – intensive BP lowering			
60–69 years old	0.52 [0.47, 0.56]		PAST-BP trial & Law et al.^9,13^
70–79 years old	0.58 [0.54, 0.63]		
80–89 years old	0.74 [0.68, 0.78]		
MI and UA – standard target			
60–69 years old	0.68 [0.65, 0.70]		PAST-BP trial & Law et al.^9,13^
70–79 years old	0.72 [0.69, 0.75]		
80–89 years old	0.78 [0.74, 0.81]		
Stroke – standard target			
60–69 years old	0.59 [0.55, 0.63]		PAST-BP trial & Law et al.^9,13^
70–79 years old	0.65 [0.61, 0.68]		
80–89 years old	0.78 [0.73, 0.82]		
**Utilities for the initial health state**			
Intensive BP lowering and standard target			
60–69 years old	0.7241	Beta	PAST-BP trial^9^
70–79 years old	0.6631	Beta	
80–89 years old	0.6362	Beta	
**Utilities for acute disease** **^b^**			
UA	0.77	Beta	NICE, Lipid Modification Guidelines^18^
MI	0.76	Beta	
Stroke	0.63	Beta	
Dead	0.00		By definition
**Utilities for long-term (chronic) disease** **^b^**			
UA	0.88	Beta	NICE, Lipid Modification Guidelines^18^
MI	0.88	Beta	
Stroke	0.63	Beta	
**Probability of death from an event**			
Fatal stroke	0.23	Beta	Bamford et al.^29^
Fatal MI			
60–69 years old	0.23		ONS, Deaths Registry 2011 & Kerr et al.^11,30^
70–79 years old	0.39		
80–89 years old	0.52		
**Annual cost of consultation per patient (UK£) – intensive BP lowering**			
GP consultations	86		PAST-BP trial & Curtis^9,16^
PN consultations	35		
**Annual cost of consultation per patient (UK£) – standard target**			
GP consultations	50		PAST-BP trial & Curtis^9,16^
PN consultations	29		
**Average cost of hypertensive drugs per patient £per year** **^c^**			
Intensive BP lowering	23		BNF 2012^2,8^
Standard target	20		
**Cost for the initial state £per year**			
Intensive BP lowering	144	Gamma	PAST-BP trial, Curtis, BNF 2012^9,16,28^
Standard target	100	Gamma	
**Costs of acute disease £one-off cost**			
Stroke	11020	Gamma	Youman et al.^19^
MI	5487	Gamma	Palmer et al.^20^
UA	3292	Gamma	Assumed 60% of MI
**Costs for long-term (chronic) disease £per year**			
Stroke	2721	Gamma	Youman et al.^19^
MI	572	Gamma	NICE, Lipid Modification Guidelines^18^
UA	572	Gamma	NICE, Lipid Modification Guidelines^18^

MI: myocardial infarction; UA: unstable angina; BP: blood pressure; GP: general practitioner; PN: practice nurse.

a Relative risk comparing blood pressure after treatment with baseline blood pressure.

b These figures are multiplied by initial health state utility to estimate new health state utility.

c Annual cost of drugs was calculated on the basis of commonest drug and dose per drug group per arm at 6 and 12 months.

### Model structure and inputs

The cohort started in the initial health state ‘previous stroke/TIA’, and a patient could remain in the ‘previous stroke/TIA’ health state if they did not have a recurrent event or died. If a cardiovascular event or death occurred the patient moved to one of four possible health states: new stroke, myocardial infarction (MI), unstable angina (UA), or dead (see Figure 1). Life tables were used to determine overall mortality dependent on age and gender, adjusted by cardiovascular disease mortality.^11^ Death was attributed to either stroke, MI or other causes. After a cardiovascular event, individuals could survive from the event or die, with death from an event occurring within a year. Individuals who survived a cardiovascular event moved to the chronic health state for that event, in which annual costs were incurred and quality of life was lower than in the ‘previous stroke/TIA’ state (Table 1). Individuals in a chronic health state were assumed to remain in that state for the rest of their lives unless they died from other causes.

Annual transition probabilities determining the risk of a cardiovascular event were based on the results of the PROGRESS trial.^12^ Age-related risk reductions for coronary heart disease (CHD) and stroke associated with subsequent reductions in SBP observed in the PAST-BP trial were obtained from Law et al. (Table 1).^13^ The risk reduction for CHD was applied to both MI and UA. This approach has previously been used by other studies to convert a decrease in SBP to reductions in CHD and stroke risk.^14,15^ The probability of each cardiovascular event occurring, the risks of dying from stroke or MI and the increased risk of death once in a chronic health state incorporated in the model are shown in Table 1. Outcomes and costs were discounted at the standard annual rate of 3.5%.^10^

### Resource use and costs

Costs are reported in UK pounds at 2011–2012 unit prices, and are discounted at 3.5% per annum.^10^

Costs were derived from a combination of standard unit costs, NHS reference costs and previously published literature and were adjusted using the Hospital and Community Health Service index to the 2011/2012 price year.^16^ Resource use and costs per patient were obtained from the PAST-BP trial and applied to the initial health state in the model.^9^ Costs for acute and chronic states were obtained from published sources.^17–20^ Costs considered over the lifetime of the model included the cost of antihypertensive drugs, consultation costs and subsequent cardiovascular events (Table 1).

### Utility values

The primary outcome measure was QALYs (Table 1). The utility value for the starting ‘previous stroke/TIA’ health state in the model was obtained from the PAST-BP trial using the overall mean EQ-5D score at baseline. The EQ-5D is a widely used generic instrument that has been validated in many patient populations, and is recommended by the National Institute for Health and Care Excellence (NICE).^10^ This was adjusted for age group using weights calculated from Ara and Brazier,^21^ which allowed a reduction in quality of life with increasing age to be incorporated in the model. Acute events were assumed to happen six months into a one-year cycle. Individuals stayed in that acute state for six months before moving into a chronic health state. Utilities for the acute state were applied mid-way through the one-year cycle and those for the chronic state at the start of the next cycle following an acute event. Future health state utilities were estimated by multiplying the starting quality of life with that of the new health state. In the base-case analysis it was assumed that different intensities of BP management had no effect on quality of life.^22^

### Analysis

An incremental cost-utility analysis was undertaken. Probabilistic sensitivity analysis was based on 10,000 Monte Carlo simulations. A gamma distribution was fitted to the costs obtained from the PAST-BP trial. Beta distributions were used to model the probability of dying from any of the cardiovascular events as well as the uncertainty around the utility values. A cost-effectiveness plane^23^ and a cost-effectiveness acceptability curve (CEAC) were constructed, the latter to depict the probability of intensive BP lowering being more cost effective compared to standard target at different cost per QALY willingness-to-pay thresholds.

Uncertainty in the results of the model was assessed through sensitivity analyses. These involved: varying the time horizon for the model; changing costs of disease and the initial cost for the intensive BP lowering arm by 30%; varying the effect size in the intensive BP lowering arm according to the 95% confidence interval of the BP reduction difference achieved at 12 months; incorporating a quality of life decrement due to antihypertensive medication by reducing utility values (multiplicatively) for the initial health state in the intensive BP lowering arm by up to 10%.^21^

## Results

The base-case lifetime costs and QALYs are presented in Table 2. Compared to a standard BP target of 140 mmHg SBP, intensive BP lowering was in a position of dominance, being cheaper and more effective. Intensive BP lowering was associated with average cost savings per patient of £169 and an additional 0.08 QALYs over 30 years.

**Table 2.** Base-case result: lifetime costs and outcomes per patient.

	Costs (£)	QALYs	Incremental cost (£)	Incremental QALYs	ICER (£per QALY)
Standard target	9889	7.4719			
Intensive blood pressure lowering	9720	7.5539	–169	0.082	Dominant

QALY: quality-adjusted life year; ICER: incremental cost-effectiveness ratio.

**Figure 2.**
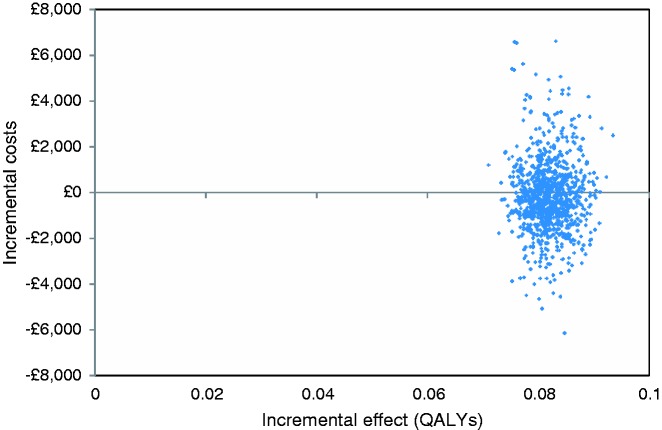
Incremental cost-effectiveness plane comparing the intensive blood pressure lowering strategy with standard target strategy or usual care.

**Figure 3.**
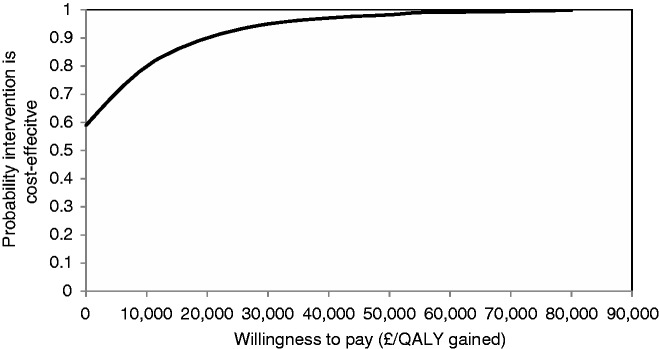
Cost-effectiveness acceptability curve for the intensive blood pressure lowering model showing the probability that the intervention is cost effective.

Figure 2 presents the cost-effectiveness plane comparing intensive BP lowering to standard target incorporating parameter uncertainty. The mean incremental costs and incremental effects (QALY gains) mostly lie in the north-east and south-east quadrants, indicating that intensive BP lowering is highly likely to be effective but with a large amount of uncertainty around its cost impact. The CEAC shows that if a decision-maker has a willingness to pay of £20,000 per QALY gained, the likelihood of cost effectiveness was 90% (Figure 3).

### Sensitivity analysis

Intensive BP lowering was cost effective at £20,000 per QALY provided at least two years of treatment was given, and became the dominant strategy after six years (Supplementary Table 1). Varying costs had little impact on the overall conclusion, but if the effect size was reduced to the lower bound of the 95% confidence interval for BP reduction, intensive targets were no longer cost effective. If intensive BP lowering is associated with a 2% or more reduction in quality of life, it is no longer effective, but remains the less expensive strategy because of the reduction in cardiovascular events. In this circumstance, the incremental cost-effectiveness ratio (ICER) suggests that standard targets are more cost effective (Supplementary Table 1).

## Discussion

We found that a strategy of intensive BP lowering in primary care, as tested by the PAST-BP trial, is likely to be cost effective. The extra initial costs of the intensive strategy are offset by subsequent cost savings in terms of reduced cardiovascular events, such that the strategy is less expensive after six years, although there was much greater uncertainty around the impact on costs as compared to the impact on benefits (Figure 2). The intensive strategy is not cost effective if it is associated with a 2% or more reduction in quality of life. However, we have found in a previous trial that reductions in BP of the order of magnitude seen in PAST-BP were not associated with any effect on quality of life,^24^ and there were no significant differences in adverse effects during the trial.^9^ This analysis assumes that the difference in BP between the arms is maintained over time: the sensitivity analysis suggested that the ICER remains below £20,000/QALY provided the time horizon is at least two years. Furthermore, there is evidence from the SPS3 trial, which involved different targets for BP in people with a history of lacunar stroke, that differences between arms were maintained up to eight years after randomisation.^25^

PAST-BP was not powered to detect differences in cardiovascular events between arms, and so the impact of observed BP reductions was estimated by applying these to the results of a systematic literature review.^13^ Recent evidence reinforces the likelihood that BP reductions are indeed likely to lead to a reduction in the risk of cardiovascular events.^6^ While this evidence was not restricted to people with previous stroke, the relative reductions in cardiovascular risk associated with reduction in BP appear to be similar in people with and without existing cerebrovascular disease.^26^

Our results are consistent with the results of a cost-effectiveness analysis based on the PROGRESS trial, which found treating people with cerebrovascular disease was cost effective, with a cost per QALY of £6927 over four years.^27^ Whereas our analysis found long-term treatment to be dominant, the PROGRESS trial found long-term treatment remained more expensive than standard care. It is likely that this difference in costs reflects changes in drug prices since the PROGRESS economic analysis was performed. Our sensitivity analysis (see Supplementary Table 1) showed that a 30% increase in the initial cost of intensive BP lowering resulted in the intensive target arm becoming more expensive than the standard care arm. A change of this magnitude is plausible given that, for example, perindopril now costs £1.72 per month, as opposed to £10.95, as applied in 2005.^27,28^

### Strengths and limitations

This study used cost and outcome data from a primary care based pragmatic randomised controlled trial in patients with a past history of stroke or TIA.^9^ The use of a Markov model overcame limitations associated with within-trial analyses, specifically allowing the modelling of effects and costs on long-term events and the assessment of the long-term cost effectiveness beyond the trial period.

The model did not include the recurrence of cardiovascular events beyond the first event. However, as the intensive lowering strategy was more effective and therefore likely to reduce cardiovascular risk, then this model simplification is likely to have produced more conservative model results.

Linked to this, an additional limitation derives from the nature of Markov models. These assume that the probability of an individual moving to any given health state in one time period depends only on their current health state. Therefore a patient’s outcomes and costs are assumed to depend only on the current health state, and this may underestimate overall costs and overestimate health outcomes for those who have suffered more than one event. Again, this is likely to have reduced the apparent cost effectiveness of intensive BP lowering.

The PAST-BP trial did not have a ‘usual care’ arm – rather it compared two active management strategies, one to an intensive target, one to a standard target. As a result, the cost-effectiveness analysis can only compare these two active strategies – it cannot examine the cost effectiveness of moving from usual care to active management.

### Clinical implications

This analysis suggests that intensive BP lowering in a post-stroke population in primary care is likely to be cost effective, despite the relatively small reduction in SBP with which it is associated. However, comparison of achieved BP in the control group with less active BP management suggests that it is also likely that active management of BP is more important than the target that is used.^9,24^ Therefore, it is difficult to determine from this economic analysis whether the priority should be to promote systolic targets less than 130 mmHg, or to promote more active management of BP. The overall conclusion from this work is that interventions lowering BP post-stroke are likely to be cost effective provided that they can be achieved without excessive additional cost or impact on quality of life. Intensive lowering of BP in primary care appears to be one such option.

## Supplementary Material

Supplementary material
