# Supplementary material for: Cost-effectiveness analysis of different systolic blood pressure targets for people with a history of stroke or transient ischaemic attack: Economic analysis of the PAST-BP study
Source: Eur J Prev Cardiol. 2016 May 25;23(15):1590–8. doi: 10.1177/2047487316651982 (PMC5030727; doi:10.1177/2047487316651982)
Supplement: Supplementary material [file Web_Table_1.pdf]

Web Table 1 Results of sensitivity analysis

	Costs (£)	QALYs	Incremental cost (£)	Incremental QALYs	ICER (£/QALY)
Varying time horizon					
20 years					
Standard target	8,962	7.1032			
Intensive BP lowering	8,794	7.1762	-168	0.0729	Dominant
10 years					
Standard target	5,092	5.1861			
Intensive BP lowering	5,012	5.2191	-80	0.0329	Dominant
7 years					
Standard target	3,387	4.0737			
Intensive BP lowering	3,362	4.0916	-25	0.0179	Dominant
6 years					
Standard target	2,786	3.6247			
Intensive BP lowering	2,779	3.6381	-7	0.0134	Dominant
3 years					
Standard target	1,270	2.0192			
Intensive BP lowering	1,286	2.0225	15	0.0034	4,590
2 years					
Standard target	834	1.3954			
Intensive BP lowering	850	1.3967	15	0.0012	11,707
1 year					
Standard target	409	0.7233			
Intensive BP lowering	419	0.7234	10	0.00007	141,231

Varying acute and chronic costs simultaneously					
30 percent decrease					
Standard target	7,173	7.4719			
Intensive BP lowering	7,177	7.5539	4	0.0820	44
30 percent increase					
Standard target	12,604	7.4719			
Intensive BP lowering	12,263	7.5539	-341	0.0820	Dominant
30 per cent increase in the initial cost for the Intensive BP lowering arm					
Standard target	9,889	7.4719			
Intensive BP lowering	10,093	7.5539	204	0.0820	2,492
Varying the intensive BP lowering arm according to the 95% CI of the BP reduction difference achieved at 12 months					
0.2 points difference					
Standard target	9,889	7.4719			
Intensive BP lowering	10,188	7.4824	299	0.0104	28,613
5.7 points difference					
Standard target	9,889	7.4719			
Intensive BP lowering	9,345	7.6125	-543	0.1406	Dominant
Reduction in quality of life due to antihypertensive medication in the intensive BP lowering arm					
1 percent reduction					

Standard target	9,889	7.4719			
Intensive BP lowering	9,720	7.4944	-169	0.0225	Dominant
2 percent reduction					
Standard target	9,889	7.4719			
Intensive BP lowering	9,720	7.4349	-169	-0.0371	** 4,552
5 percent reduction					
Standard target	9,889	7.4719			
Intensive BP lowering	9,720	7.2562	-169	-0.2157	** 782
10 percent reduction					
Standard target	9,889	7.4719			
Intensive BP lowering	9,720	6.9584	-169	-0.5135	** 328

** These positive ICERs represent points in the south-west quadrant of the incremental cost-effectiveness plane: they indicate a cost saving accompanied by a loss of QALYs. In each case, the ICER is below all recognised thresholds: if these were to be the true values, this would indicate that the cost saving was not worth making.
